# Improved mould resistance and antibacterial activity of bamboo coated with ZnO/graphene

**DOI:** 10.1098/rsos.180173

**Published:** 2018-08-15

**Authors:** Jin Wang, Jingpeng Li, Xiaowei Zhuang, Xin Pan, Haixia Yu, Fangli Sun, Jiangang Song, Chunde Jin, Yingti Jiang

**Affiliations:** 1 Zhejiang Provincial Key Laboratory of Biological and Chemical Utilization of Forest Resources, Zhejiang Academy of Forestry, Hangzhou 310023, People's Republic of China; 2 China National Bamboo Research Center, Chinese Academy of Forestry, Hangzhou 310012, People's Republic of China; 3 School of Engineering, Zhejiang Agricultural and Forestry University, Lin'an 311300, People's Republic of China; 4 Zhejiang Yongyu Bamboo Joint-Stock Co., Ltd, Anji 313301, People's Republic of China

**Keywords:** bamboo, graphene, zinc oxide, mould resistance, antibacterial activity

## Abstract

Bamboo is susceptible to mould and attack by fungi because of its high content of starch and sugar. To make bamboo-based outdoor materials, a new type of bamboo timber with improved mould resistance and antibacterial activity, coated with reduced graphene oxide and nanocrystal ZnO (abbreviated as RGO@ZnOBT), was fabricated by a two-step dip-dry and hydrothermal process. A possible synthesis mechanism for RGO@ZnOBT was investigated by X-ray diffraction, scanning electron microscopy, transmission electron microscope, energy-dispersal X-ray analysis, X-ray photoelectron spectroscopy and Fourier-transform infrared spectroscopy. According to the China standard test method, the *Aspergillus niger* mould resistance of RGO@ZnOBT is grade 2, whereas the *Trichoderma viride* and *Penicillium citrinum* mould resistance of RGO@ZnOBT is grade 0, both of which are better than the grade 4 of original bamboo timber. The *Escherichia coli* resistance test showed that the antibacterial circle of RGO@ZnOBT is 3 mm, which is significantly higher than that of original bamboo timber (0 mm). The antibacterial activity of treated bamboo is significantly improved compared with that of untreated bamboo.

## 1. Introduction

Because of rapidly growing economies, the infrastructure of cities is being continuously improved in terms of the human living environment. In this regard, outdoor spaces are now attracting a wider audience [1], and there is a great demand for outdoor wood-based furniture in the market. Bamboo, because of its rapid growth rate, excellent specific strength and wide availability, has become more and more popular as an outdoor furniture material in the future development of the forestry industry [2]. A survey of the bamboo industry shows that the number of outdoor bamboo products has gradually increased in recent years. However, bamboo itself is a natural organic material which is rich in protein, carbohydrate and other nutrients and is prone to mildew, being eaten by moths and rotting. Therefore, bamboo outdoor products must undergo fungus-resistant treatment according to their varying environmental applications [3].

There are two main types of protection for bamboo products: chemical modification, such as chemical preservative treatment, and physical modification, such as heat treatment [4–6]. In recent years, many investigators have successfully developed wood preservatives containing copper, chromium and arsenic (CCA) as the active ingredient [7,8]. However, CCA-treated wood or bamboo outdoor products will easily cause pollution to the environment [9,10].

Graphene is a monolayer of carbon atoms that are tightly packed into a two-dimensional crystal [11]. Hu *et al*. [12] have reported a macroscopic antibacterial graphene-based paper, which demonstrates that graphene materials with a superior ability to inhibit bacterial growth may be used for promising environmentally friendly application. Liu *et al*. [13] compared the antibacterial activity of four types of graphene-based materials including graphite (GT), graphite oxide (GTO), graphene oxide (GO) and reduced graphene oxide (RGO) in a bacterial model of *Escherichia coli* and found that, under similar concentration and incubation conditions, GO dispersion showed the highest antibacterial activity, sequentially followed by RGO, GT and GTO. We adopted this method by using GO dispersion to treat bamboo, and the results showed that the decay resistance of bamboo timber was improved [14]. Also, we fabricated a superamphiphobic surface on the bamboo surface with ZnO nanoparticles [15]. Nano-sized ZnO exhibited varying morphologies and showed significant antibacterial activity over a wide spectrum of bacterial species explored by a large body of researchers [16–18]. ZnO is currently being investigated as an antibacterial agent in both microscale and nanoscale formulations [19].

Both graphene-based material and microscale/nanoscale ZnO have antimicrobial properties. Therefore, in this work, combined with first an RGO coating as the middle layer and then nano-sized zinc oxide as the outer layer, a new mixed coating on a bamboo surface was prepared. The as-prepared bamboo samples after test showed improved mould resistance and antibacterial activity.

## 2. Material and methods

### 2.1. Materials

Graphite powder (less than 20 µm) was provided by Shanghai Boyles Chemical Co. Ltd; sulfuric acid, hydrochloric acid, nitric acid, potassium permanganate, zinc acetate, zinc nitrate, hydrogen peroxide, methanol, ethanol, ammonia, monoethanolamine and hexamethylenetetramine (HMTA) were provided by Huipu Chemical Company (Hangzhou, Zhejiang, China) and used without further purification. *Trichoderma viride*, *Pencillium citrinum*, *Aspergillus niger* and *E. coli* were provided by the Beijing North Carolina Souren Biotechnology Research Institute. The bamboo specimens were processed into blocks with dimensions of 50 × 20 × 5 mm (longitudinal × tangential × radial), which were then cleaned by washing sequentially with deionized water and then ethanol for 30 min, and finally dried under vacuum at 50°C for 24 h. The original bamboo timber subjected to the above process is abbreviated as BT.

### 2.2. Preparation of RGO@ZnOBT samples

**Figure 1.**
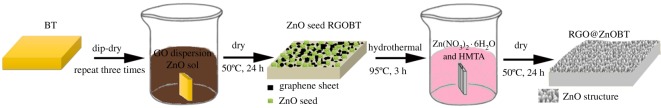
Schematic illustration of the synthetic process of RGO@ZnOBT.

GO was synthesized from graphite powder according to the modified Hummer's method [20]. A 2 mg l^−1^ GO solution was obtained by adding distilled water to the resulting GO powder and then sonicated to achieve a uniform GO dispersion. A transparent homogeneous ZnO solution was achieved by mixing 200 ml of 0.5 M zinc acetate methanol solution and 200 ml of ethanolamine solution at 60°C and stirring for 30 min. The GO dispersion and the ZnO solution were mixed with a volume ratio of 1 : 9. The bamboo samples were subsequently dipped into the above mixed solution for 10 min and then dried at 140°C for 10 min. The dip-dry process was repeated three times. After that, the treated bamboo samples were dried at 50°C for 24 h under vacuum. Bamboo samples with GO and ZnO seed coating were transferred into a Teflon-lined stainless steel autoclave. An equal volume of 0.03 M Zn(NO_3_)_2_·6H_2_O and 0.03 M HMTA was added to the autoclave. The autoclave was sealed and maintained at 95°C for 3 h. After the hydrothermal reaction, the treated bamboo samples were removed and rinsed with deionized water and then dried at 50°C for 24 h under vacuum. The as-prepared samples of non-crystalline ZnO are abbreviated as RGO@ZnOBT and the whole synthesis scheme is shown in figure 1.

### 2.3. Characterization

The surface morphologies of the samples were characterized by scanning electron microscopy (SEM; Quanta 200; FEI, USA). The surface chemical compositions of the samples were determined via energy dispersive spectroscopy (EDX, attached to the SEM). Transmission electron microscopy (TEM) experiments were performed on a Tecnai G_20_ electron microscope (FEI, USA) with an acceleration voltage of 200 kV. Carbon-coated copper grids were used as the sample holders. The crystalline structures of the samples were identified by X-ray diffraction (XRD; D/MAX 2200; Rigaku, Japan) using Cu K_α_ radiation (*λ* = 1.5418 Å) at a 2*θ* scan rate of 4° min^−1^, 40 kV, 40 mA, ranging from 5° to 80°. The presence of functional groups in the samples was confirmed through Fourier-transform infrared (FTIR) spectroscopy (Spectrum One; PerkinElmer, USA). The surface elemental composition analyses were conducted based on X-ray photoelectron spectroscopy (XPS; Scientific-K-Alpha 1063; Thermo Fisher, UK) with an Al Kα monochromatic X-ray source, in which all of the binding energies were calibrated with reference to the C 1s peak (284.8 eV).

### 2.4. Mould-resistant test

**Figure 2.**
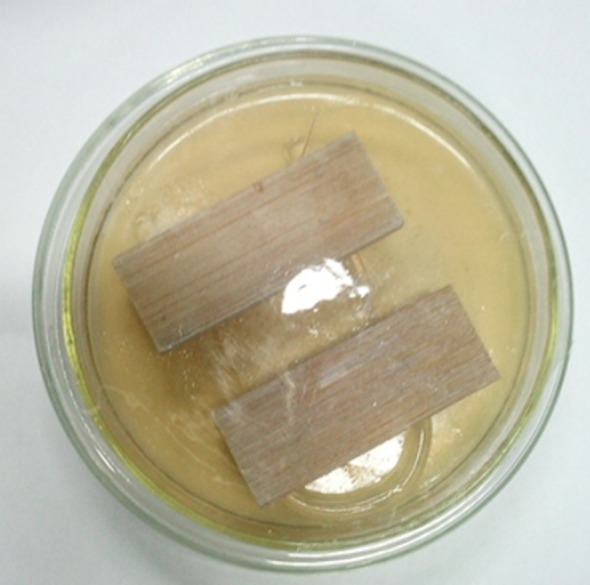
Method for mould test.

Fungus-resistant tests were conducted in accordance with the Standard Method for Testing Fungicides for Controlling Sapstain and Mould on Unseasoned Lumber and the Testing Method for Anti-mould Chemicals in Controlling Mould and Blue Stain Fungi on Wood. Three kinds of mould fungi including *T. viride*, *P. citrinum* and *A. niger* were applied in the mould resistance test. Under aseptic conditions, the mycelium and spores were inoculated into sterile water by an inoculation ring and the mixed bacterial suspension was used for inoculation. About 0.2 ml of bacterial suspension was uniformly streaked onto the Petri dishes filled with potato dextrose agar (PDA) substrate. The Petri dishes were then placed into an incubation cabinet, and maintained at a temperature of 26°C and relative humidity of 92% for one week. Then, the fungi in the Petri dishes that had grown were used to inoculate the specimens. Before inoculation, a sterilized U-shaped glass rod (3 mm in diameter) was placed onto the PDA substrate which was covered with mycelium, and two specimens were collected separately onto each glass rod (figure 2). Six specimens (size 50 × 20 × 5 mm) of RGO@ZnOBT and BT for three repeated tests were used for each fungus. After inoculation, the dishes were moved into an incubation cabinet, and maintained at a temperature of 26°C and relative humidity of 92% for 30 days. According to the evaluation standard in table 1, the infection area was recorded and the average value of three repeated tests was set as the final result.

**Table 1.** Grades of infection.

infection value	mould coverage on specimens
0	the surface of specimens have no mycelium
1	the area of mould infection <1/4
2	the area of mould infection 1/4–1/2
3	the area of mould infection 1/2–3/4
4	the area of mould infection >3/4

### 2.5. Antibacterial test

Antimicrobial tests were conducted by the bacterial inhibition ring method (agar plate diffusion test/CEN/TC 248 WG 13) and the reduction in bacterial growth test (EN ISO 20743: 2007 Transfer Method). *E. coli* (ATCC25923, Gram-negative bacterium) were applied in the antibacterial test. The freeze-dried bacteria were activated in nutrient broth at 37°C for 24 h. The agar medium was then poured into the Petri plates and cooled under a laminar airflow. Approximately 105 colony-forming units of *E. coli* were inoculated on each plate. The BT and RGO@ZnOBT samples were processed with a diameter of 5 mm and thickness of about 2 mm, and then planted onto the agar plates. After incubation at 37°C for 24 h, the diameter of inhibition ring was measured in all three repeated tests and the average value was set as the final result.

## 3. Results and discussion

### 3.1. Characterization of bamboo timber and RGO@ZnOBT

**Figure 3.**
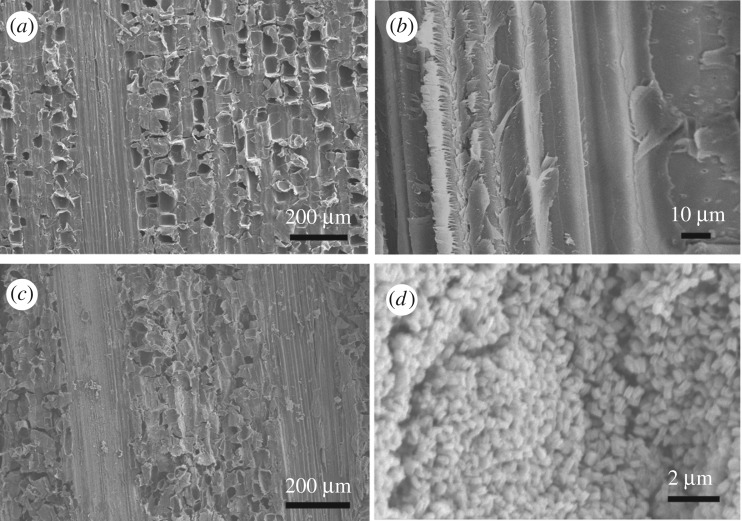
Typical low- and high-magnification SEM images of BT (*a*,*b*) and RGO@ZnOBT (*c*,*d*).

Figure 3 shows the typical low- and high-magnification SEM images of BT and RGO@ZnOBT. Figure 3*a*,*b* shows that the original bamboo surface is very clean and that there are no other hybrids on the bamboo vessels and fibres. The nanoparticles with a diameter of 200–300 nm can be seen on the bamboo surface in figure 3*c*,*d*. In our previous study, the bamboo surface was coated with RGO nanosheets in the first step with hydrothermal treatment by graphene oxide [21]. The reason for this is that the RGO coating can provide a more combined position for the location of ZnO seeds. In this study, after the first step of three repeats of the dip-dry process, more ZnO seeds were planted on the bamboo surface. In the second hydrothermal process, ZnO nanocrystals were grown and formed spherical ZnO nanoparticles. More and more ZnO nanocrystals from the hydrothermal solution gathered together on the bamboo surface and formed spherical particles with larger size. As a result, the bamboo surface was fully coated with ZnO nanocrystals.

**Figure 4.**
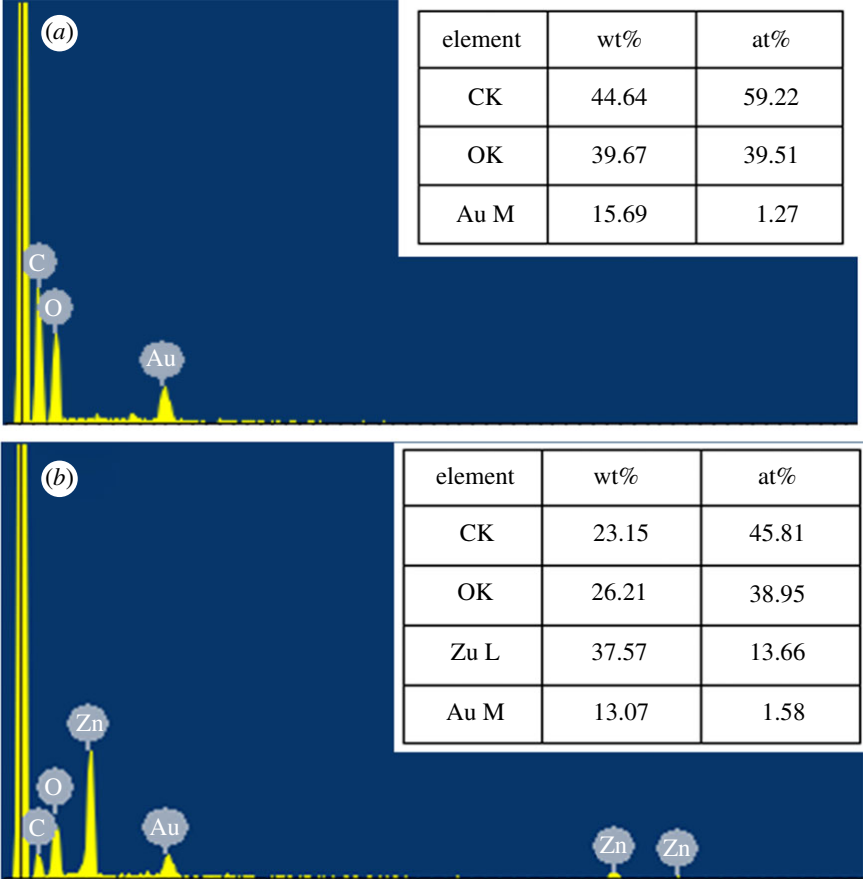
EDX spectra and element contents of BT (*a*) and RGO@ZnOBT (*b*).

 Figure 4*a*,*b* shows the chemical element components and contents of BT and RGO@ZnOBT. C, O and Au elements were detected in both of the two bamboo samples, and elemental Zn was only detected in RGO@ZnOBT. SEM showed that, in the pre-treatment process, the Au element was coated for electric conduction [22]. C and O elements were from the original bamboo substrate [23]. Elemental Zn could be detected in RGO@ZnOBT samples as shown in figure 4*b*. Because the ZnO nanocrystals are composed of Zn and O elements, the elemental Zn content in RGO@ZnOBT was higher than that in BT. The results proved that ZnO was successfully loaded onto the bamboo surface.

**Figure 5.**
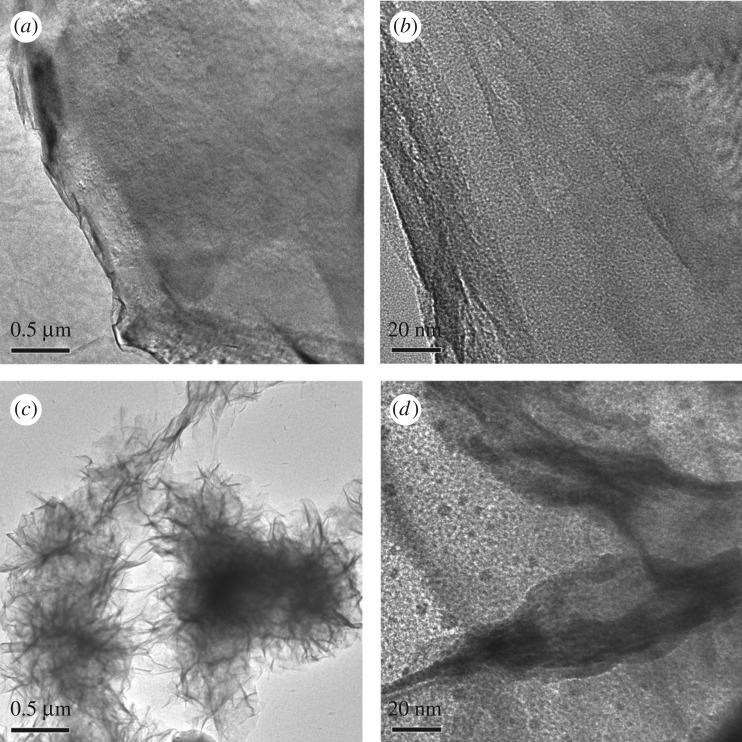
Typical low- and high-magnification TEM images of the GO dispersion (*a*,*b*) and the RGOBT–ZnO reaction solution after the hydrothermal step.

Typical low- and high-magnification TEM images of the graphene dispersion and the RGOBT–ZnO reaction solution after the hydrothermal process are shown in figure 5. The GO sheets of 1–5 µm diameter and a single layer are observed clearly in figure 5*a*,*b*. There are obvious folds within the transparent GO sheets. In the dip-dry process, GO sheets combined with the bamboo surface and then were reduced. Meanwhile, the ZnO seeds were loaded onto the graphene network, especially in the folds of the RGO sheets. ZnO particles with diameter 0.5–1 µm and bamboo nutrients such as starch granules can be seen in figure 5*c*,*d*. As a result of the hydrothermal treatment, ZnO crystals were loaded onto the bamboo surface and autoclave bottom, so the solution had a low ZnO crystal content. Meanwhile, nutrients such as starch granules were boiled out and remained in the solution.

**Figure 6.**
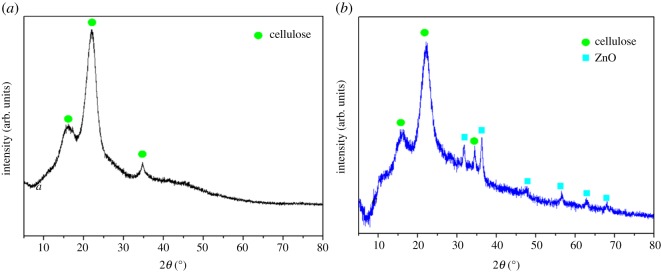
XRD pattern on BT (*a*) and RGO@ZnOBT (*b*).

Figure 6 shows the XRD patterns of BT and RGO@ZnOBT. The original bamboo showed typical peaks at 2*θ* of 15.7°, 22.3° and 34.8°, corresponding to the cellulose [24]. The RGO@ZnOBT sample showed typical peaks at 2*θ* of 34.4°, 36.3°, 47.5°, 56.5°, 62.8° and 68.0°, corresponding to the (002), (101), (102), (110), (103) and (200) crystal plane of ZnO, respectively (JCPDS no. 89-0511) [25,26]. There were no other peaks detected, which indicated that only high purity ZnO nanocrystals with hexagonal wurtzite structure were present, proving that ZnO nanocoatings attached onto the RGO layer were highly crystalline.

**Figure 7.**
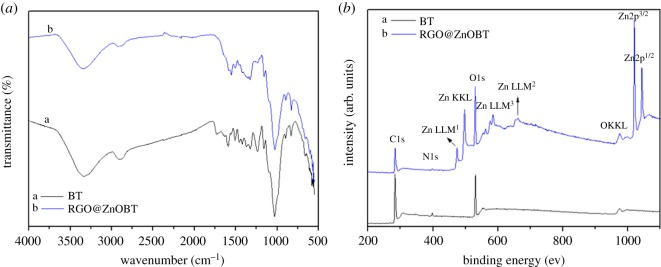
FTIR spectra (*a*) and XPS spectra (*b*) of BT and RGO@ZnOBT.

Figure 7*a* shows the FTIR spectra of BT and RGO@ZnOBT. There are obvious absorption peaks at 1728, 1620, 1407, 1221 and 1051 cm^−1^ for GO [27]. However, when BT was coated with a graphene layer and a ZnO layer, the intensity of the absorption peaks in 1728, 1620, 1407 and 1221 cm^−1^ significantly decreased. The results proved that GO was reduced by the dip-dry process and graphene has no obvious characteristic peak of C=O, C=C, C–OH and C–O [28]. The intensity of the absorption peak at 1051 cm^−1^ increased, implying more alkoxy stretching vibration of C–O occurred in RGO@ZnOBT. This can be explained as follows: when the RGO network was coated with more ZnO nanocrystals, ZnO and graphene combined with C–O bonds. The absorption peak of O–H in the broad band was lower in RGO@ZnOBT than in BT, which showed that, when coated with ZnO nanocoating, the O–H groups decreased.

Here, XPS was used to characterize the chemical composition of the BT and RGO@ZnOBT. As shown in figure 7*b*, 10 peaks of C1s, N1s, O1s, OKKL, Zn2p^3/2^, Zn2p^1/2^, Zn KKL, Zn LLM^1^, Zn LLM^2^ and Zn LLM^3^ FKLL could be clearly observed in the spectrum of RGO@ZnOBT. BT only showed the C, O and N elements. RGO@ZnOBT mainly contained C, O, N and Zn elements. ZnO appeared with a number of characteristic peaks, such as Zn2p^3/2^, Zn2p^1/2^, ZnKKL, ZnLLM^1^, ZnLLM^2^ and ZnLLM^3^, and the maximum peak appeared at 1021.5 eV, corresponding to the Zn2p^3/2^ peak. The results are consistent with the EDX analysis. Because of RGO being the middle layer, ZnO nanocrystals had a higher chance of forming on the bamboo surface and produced a more dense coating to protect the bamboo surface.

### 3.2. Mould-resistant property

**Figure 8.**
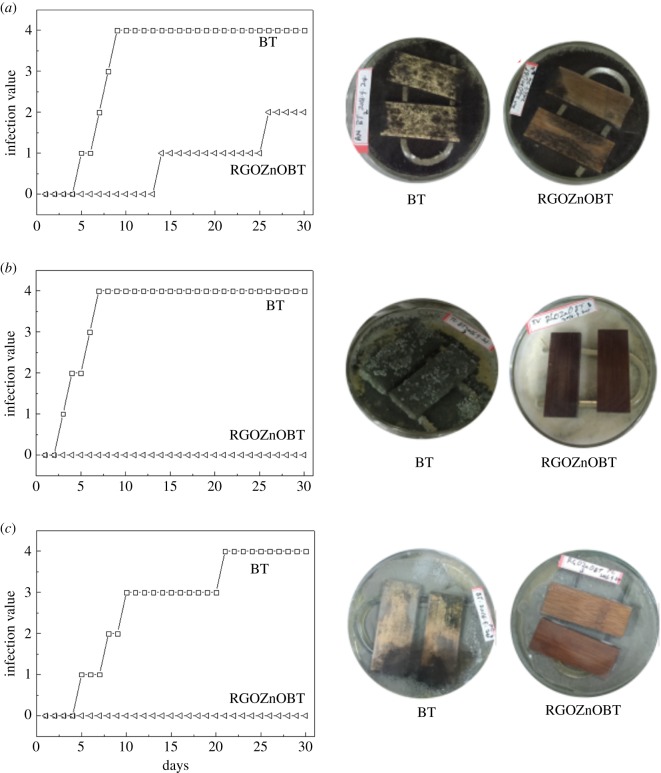
Mould resistance of BT and RGO@ZnOBT against *A. niger* (*a*), *T. viride* (*b*) and *P. citrinum* (*c*); and the digital photos of the mould resistance test.

Figure 8*a*–*c* shows the mould resistance of BT and RGO@ZnOBT against *A. niger*, *T. viride* and *P. citrinum*. From the digital photos of bamboo samples in figure 8, the original bamboo surface is very clean and has no mycelium; the RGO@ZnOBT surface has a white layer of ZnO nanocrystals. Both the BT and RGO@ZnOBT were inoculated with the different mould fungi for 30 days; the infection area ratios of the bamboo surface were analysed. As shown in figure 8*a*, original bamboo was 100% infected with the mycelium of *A. niger*, which was classed as grade 4 according to table 1. However, because of the protection of the ZnO coating, the RGO@ZnOBT samples have no mycelium in the first 13 days, and less than 50% infection area was observed after 30 days, which was classed as grade 2. So, the new bamboo samples with graphene/ZnO coating had improved mould resistance against *A. niger*, which can be seen directly from the digital photos in figure 8.

Using the same method, figure 8*b*,*c* shows that bamboo coated with graphene/ZnO coating had excellent mould resistance. For the *T. viride* infection, original bamboo samples had a ratio of 100% infection after 7 days, compared with no mycelium (grade 0) on the RGO@ZnOBT surface after 30 days. For the *P. citrinum* infection, original bamboo samples needed a longer time (21 days) to cover the whole surface. Also, no mycelium could be seen on the RGO@ZnOBT surface, which showed that the RGO@ZnOBT has a mould resistance grade of 0 against *P. citrinum*. The effects of the graphene/ZnO coating on inhibiting fungal growth were significantly different. The as-prepared bamboo coating material was more effective on *T. viride*/*P. citrinum* than on *A. niger*.

In summary, bamboo coated with graphene/ZnO possesses improved mould-resistant properties. Bamboo is easier to mould than wood because of its higher content of starch and sugar. Using the method to coat bamboo with graphene/ZnO has a promising future for making outdoor bamboo products.

### 3.3. Antibacterial activity

**Figure 9.**
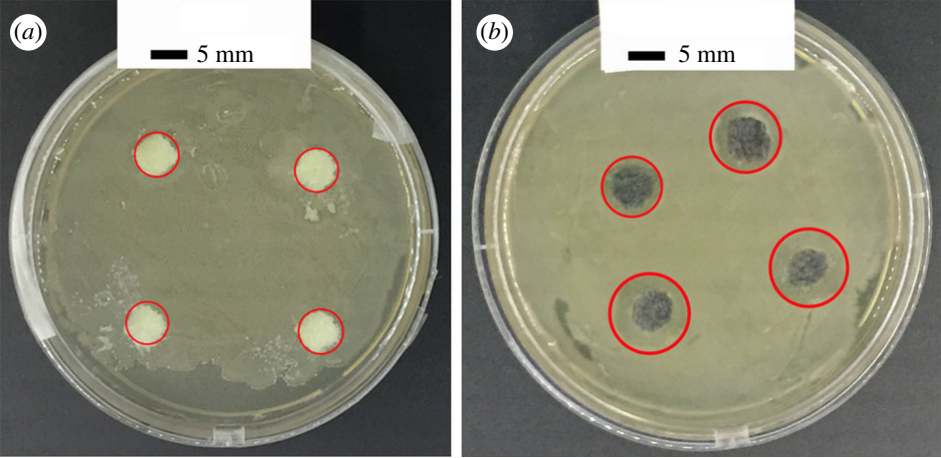
Antibacterial activity of (*a*) BT and (*b*) RGO@ZnOBT against *E. coli*.

Figure 9 shows the antibacterial test result of BT and RGO@ZnOBT against *E. coli*. Under the same conditions of incubation for 24 h at 37°C, the *E. coli* grew well in the BT and RGO@ZnOBT samples. Figure 9*a* shows that the original bamboo, which is used as the control group, does not show any antibacterial activity against *E. coli*. The graphene/ZnO-coated bamboo could inhibit the growth of *E. coli* around the sample and a distinct zone of inhibition can be seen clearly. The width of the inhibition zone of the original bamboo samples was 0 mm, as shown in figure 9*a*. The average width of the inhibition zone of the RGO@ZnOBT samples was 3 mm, as shown in figure 9*b*. The observed zone of inhibition is the result of leaching of active biocidal ZnO particles that were present in the bamboo into the surrounding aqueous medium. This proves that bamboo samples coated with graphene/ZnO material exhibit improved antibacterial ability against *E. coli*.

### 3.4. Synthesis mechanism and antimicrobial mechanism

According to the characterization, the chemical mechanism can be explained as follows: in the first step, zinc acetate provides the required Zn^2+^ ions, then the Zn^2+^ ions react with the OH^−^ ions from the alkaline solution to form ${\left[\text{Zn}{\left(\text{OH}\right)}_{4}\right]}^{2-}$; in the second step, ${\left[\text{Zn}{\left(\text{OH}\right)}_{4}\right]}^{2-}$ breaks down into ZnO and forms a nanocoating by continuous crystallization [29]. The reaction process can be described as follows [30]:

3.1 $$\text{Z}{\text{n}}^{2+}+4\text{O}{\text{H}}^{-}\to {\left[\text{Zn}{\left(\text{OH}\right)}_{4}\right]}^{2-}$$

and

$${\left[\text{Zn}{\left(\text{OH}\right)}_{4}\right]}^{2-}\to \text{ZnO}+{\text{H}}_{2}\text{O}+2\text{O}{\text{H}}^{-}$$

Combined with the preparation process in figure 1, a possible synthesis mechanism can be conducted as described below.

When original bamboo is immersed in GO dispersion and ZnO solution, the internal spaces of the bamboo can be filled with GO dispersion; meanwhile, the bamboo surface can absorb the GO and ZnO seeds. At 140°C for 10 min in the dip-dry process, GO is reduced, and the RGO nanosheets are coated via layer-by-layer self-assembly [14]. By repeating the dip-dry step, the bamboo surface can be coated with more and more RGO sheets and ZnO seeds. In the hydrothermal process, the ZnO crystals were formed via the above chemical reaction shown in steps (3.1) and (3.2), and were deposited on the outer layer of the bamboo surface. The ZnO nanocrystals were loaded onto the whole bamboo surface and formed spherical particles with a diameter of 200–300 nm, according to the SEM results. The ZnO layer and graphene medium layer combined together via the C–O bonds and physical adsorption, which was shown in the XRD, FTIR and XPS analysis.

The stability of the graphene/ZnO coating should be a concern. In our previous study, we adapted the method by using only ZnO coating or RGO coating on the bamboo surface. The durability of ZnO bamboo or RGO bamboo showed that after 3 h of boiling in water or after being washed by rain for 10 days in an outdoor field, the ZnO or graphene coating could not be easily washed away and showed excellent durability [14,15]. By using a graphene/ZnO coating, inside the RGO@ZnOBT samples, the cell spaces were filled with GO, and the outer layer was deposited with ZnO nanoscale particles. According to the previous study of ZnO bamboo or RGO bamboo, it can be inferred that the graphene/ZnO coating cannot be easily washed away.

In outdoor use, bamboo timber will be exposed for long periods under different climatic conditions to many moulds, fungi and bacteria. To ensure the service life of outdoor bamboo products, the chemical substances with antimicrobial activity should be loaded onto the bamboo surface and permeated into the bamboo cell spaces. In the dip-dry process, GO absorbed in the cell gaps could combine with the chemical composition of the bamboo cell wall. In the hydrothermal process, ZnO nanocrystals were deposited on the bamboo surface. Therefore, the graphene/bamboo coating treatment can improve the antimicrobial properties of the surface and the interior of bamboo timber.

The antimicrobial activity of RGO@ZnOBT is attributed to the graphene material and ZnO nanoparticles. The antimicrobial mechanism of ZnO nanoparticles can be explained by: (i) electrostatic forces of physical interactions; (ii) oxidative stress of the cell membranes; (iii) photocatalytic generation of H_2_O_2_, and (iv) disorganization of the bacterial membrane [31–33]. It has been reported that GO and RGO exhibit strong antibacterial activity. The antimicrobial mechanism can be explained by: (i) membrane stress induced by the sharp edges of the graphene nanosheets and (ii) oxidative stress on neural phaeochromocytoma-derived PC12 cells [34,35]. These mechanisms of graphene and ZnO nanoparticles complemented each other and resulted in the significant antimicrobial activity of the RGO@ZnOBT, which led to improved mould resistance and antibacterial ability. The interactions between the graphene/ZnO materials and the bacteria are mostly toxic, when compared with only using graphene or ZnO nanoparticles. The graphene/ZnO-coated bamboo will be exploited for antimicrobial applications such as in the green landscaping industry.

## 4. Conclusion

A new type of bamboo timber coated with graphene/ZnO was fabricated by a two-step dip-dry and hydrothermal process. According to the China test standards, the *A. niger* mould resistance of RGO@ZnOBT reached grade 2, and both the *T. viride* and *P. citrinum* mould resistance of RGO@ZnOBT reached grade 0. Bamboo coated with graphene/ZnO possesses improved mould-resistant properties and antibacterial activity. The possible synthesis mechanism and antimicrobial mechanism of RGO@ZnOBT were discussed.

## Data accessibility

Our data are deposited at Dryad: http://dx.doi.org/10.5061/dryad.447b022 [36].
